# Driving-Simulator-Based Test on the Effectiveness of Auditory Red-Light Running Vehicle Warning System Based on Time-To-Collision Sensor

**DOI:** 10.3390/s140203631

**Published:** 2014-02-21

**Authors:** Xuedong Yan, Qingwan Xue, Lu Ma, Yongcun Xu

**Affiliations:** MOE Key Laboratory for Urban Transportation Complex Systems Theory and Technology, Beijing Jiaotong University, Beijing 100044, China; E-Mails: 12120932@bjtu.edu.cn (Q.X.); lma@bjtu.edu.cn (L.M.); 12114242@bjtu.edu.cn (Y.X.)

**Keywords:** red-light running, audio warning information, driving simulator, driving behavior, structural equation modeling

## Abstract

The collision avoidance warning system is an emerging technology designed to assist drivers in avoiding red-light running (RLR) collisions at intersections. The aim of this paper is to evaluate the effect of auditory warning information on collision avoidance behaviors in the RLR pre-crash scenarios and further to examine the casual relationships among the relevant factors. A driving-simulator-based experiment was designed and conducted with 50 participants. The data from the experiments were analyzed by approaches of ANOVA and structural equation modeling (SEM). The collisions avoidance related variables were measured in terms of brake reaction time (BRT), maximum deceleration and lane deviation in this study. It was found that the collision avoidance warning system can result in smaller collision rates compared to the without-warning condition and lead to shorter reaction times, larger maximum deceleration and less lane deviation. Furthermore, the SEM analysis illustrate that the audio warning information in fact has both direct and indirect effect on occurrence of collisions, and the indirect effect plays a more important role on collision avoidance than the direct effect. Essentially, the auditory warning information can assist drivers in detecting the RLR vehicles in a timely manner, thus providing drivers more adequate time and space to decelerate to avoid collisions with the conflicting vehicles.

## 1. Introduction

With the rapid growth of the number of passenger cars, traffic safety-related issues have attracted worldwide attention. For example, in 2001 a goal for reducing road accidents was set up by European countries [1]. Among all types of traffic accidents, red-light running (RLR) has been identified as a major cause of crashes occurring at intersections, and it was estimated to account for 20% of all accidents [2]. In addition, the severity level of accidents caused by RLR seems to be higher [3]. More specifically, Retting *et al.* [4] found that the injury rate of drivers in RLR accidents is 47%, whereas the rate in other types of accidents is only 33%. The RLR collisions typically occur between legal vehicles traveling during the green light phase and the illegal RLR vehicles unexpectedly crossing the intersections from the conflict directions.

Due to the heavy damage produced by intersection collisions, the concept of intersection collision avoidance systems (ICAS) has emerged to solve this issue of traffic safety. Three representative initiatives that provide ICAS solutions were organized by the U.S. Intelligent Transportation Systems Joint Program Office, namely the Intelligent Vehicle Initiative (IVI), the Vehicle Infrastructure Integration (VII) initiative, and the Cooperative Intersection Collision Avoidance System (CICAS) Program. Through these programs, the potential ICAS will use both vehicle-based and infrastructure-based technologies to help drivers approaching intersections understand the state of activities occurring in that location [5]. Intersection collision warning systems (ICWS) which can provide warning information to the drivers entering the intersection have been widely used to facilitate collision avoidance. Generally, the intersection collision warning system (ICWS) can detect the approaching vehicles and evaluate their arrival time to an intersection with sensors in the vehicles and devices located at the intersection [6].

Studies which were focused on the key technologies [7,8] and the structural design [9–11] of ICWS have been conducted recently. For instance, Yang *et al.* [12] designed a kind of intersection collision warning system using wireless communication technology which is capable of estimating specific collision points according to the geometric positions, directions, speed and other parameters of vehicles. Then, the warning information will be delivered to drivers in case of potential collisions by the system. Dedicated short-range communication technology has been accepted as one of the wireless technologies most suitable for automatic crash prevention because it enables direct vehicle-to vehicle and vehicle-to-infrastructure communications in very short time frames [13]. The emergence of vehicle infrastructure integration (VII) provides ideas and methods designed specifically for reducing the chance of being involved in RLR collisions. With such a system, warning technologies for addressing RLR collision problems are being developed, which allow drivers to monitor the road situation by means of a series of detection and sensing devices. If the system detects any possible RLR vehicles, warning information is then posted to drivers in order to promote their attention to the hazards from RLR vehicles. The posted warning information is committed to provide effective suggestions and thus assist drivers in taking appropriate response actions for avoiding the potential collisions with the RLR vehicles. Nekoui *et al.* [14] presented a design of intersection collision warning systems based on VII. The systems consist of roadside and on-board units, in which appropriate alarm messages are disseminated by the roadside unit. It was designed to predict a potential collision at the intersection and notify endangered vehicles of the moving car which is about to cross the red light. The experimental results showed that the system is effective at avoiding RLR collisions.

However, the ICWS on-road tests with sensors equipped in vehicles and devices at intersections, require a detecting radar be set up by the roadside and sensors be installed in all vehicles that pass the intersection. Thus, there was a high requirement for a stable and reliable manner to send warning signals properly [15]. Additionally, one of the impediments of experimental research of RLR warning technologies is that the pre-crash scenarios are dangerous for subjects due to the involved high-risk driving activities. Those concerns create many difficulties and make an on-road tests very complex. With such consideration, it was suggested that RLR collision experiments would be conducted using driving simulators which have advanced features in light of simpler operations, higher safety conditions and lower cost [16,17].

The warning forms used by researchers using simulating driving can be categorized into visual warnings, vibratory warnings, tactile warnings and auditory warnings. Experiment made by Werneke and Vollrath [18] showed that appropriate visual warning signals could improve driving behavior by shortening drivers' reaction times. The experiment conducted by Cristy *et al.* [19] studied the effectiveness of intuitive vibratory warning signals using a driving simulator and showed that drivers react faster, and the safety distance appeared to be larger with the help of warning signals. The results indicated that vibratory warning signals are effective at calling drivers' attention to potential collisions. Tactile warnings were also found to be an effective in-vehicle countermeasure to alert drivers to pay attention to traffic crash risks [20,21]. Scott and Gray [22] made a comparison of tactile, visual and auditory warnings, and found that tactile warnings improved driver braking response better than visual and auditory warnings. As to the auditory display method, Chang *et al.* [16] compared beep sounds and speech messages to alert the RLR vehicle. Shorter response times and slower speeds were found with an installed warning system in the vehicle. Furthermore, shorter response time and slower speeds were inclined to lead to lower collision rates, which were 16% and 26% with a beep warning and a speech warning, respectively. Inman *et al.* [17] proposed a concept of RLR vehicle warning system for the purpose of detecting the RLR vehicles and providing warning information for drivers. In more detail, the warning information is delivered to vehicles which are in the green phase in the format of flashing signs or lights in vehicles. Based on the driving simulator test results, once the subjects had noticed the warning information, most of them chose to apply the brakes and were more likely to successfully avoid collisions from RLR vehicles. Although the driving-simulator-based studies have been conducted for the developments and tests of RLR vehicle collision warning technologies, the research scope of auditory warnings for RLR vehicles is still limited and the detailed characteristics of driving behavior under the warning system still need to be comprehensively understood. Especially, there is a lack of literature which focuses on examining the differences between the effects of directional and undirectional information on driving behavior and crash avoidance performance. Also, no previous investigation of the causal relationships between warning conditions, human factors, driving behavior and the effectiveness of collision avoidance has been undertaken. Because driving simulators are able to establish a wide variety of RLR scenarios for comparison purposes as well as enriching the test results, this study utilized a high-fidelity driving simulator to simulate the pre-crash scenarios of RLR collisions and then investigated the impact of audio warning information on crash avoidance driving behavior. The goal of this study is to test the working function and mechanism of an in-vehicle speech collision avoidance warning system under different warning conditions issued by a Time-To-Collision (TTC) sensor.

## 2. Methodology

### 2.1. Apparatus

**Figure 1.**
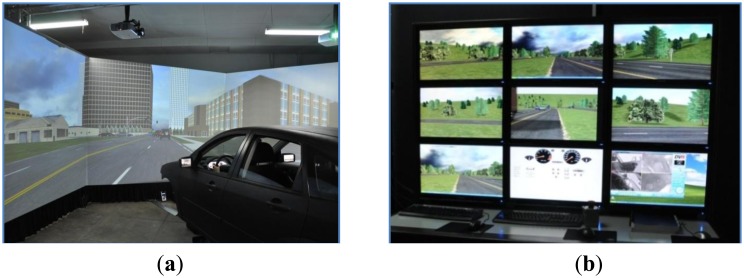
Illustration of the driving simulator system; (**a**) Driving simulator. (**b**) Monitoring and controlling systems.

**Figure 2.**
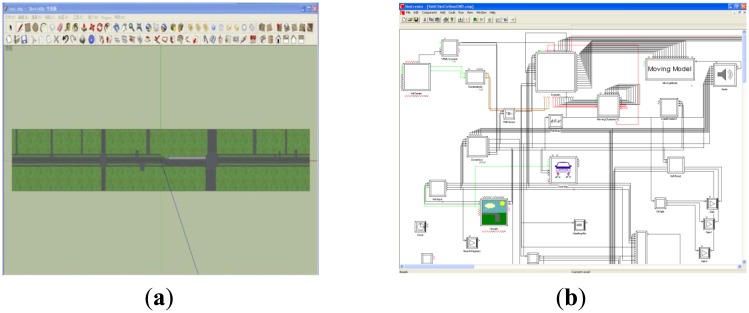
Interface of the simulator software; (**a**) SimVista. (**b**) SimCreator.

The driving simulator used for this study is able to simulate various road traffic environments and driving scenes, as shown in Figure 1. The vehicle cockpit in the simulator is designed in full accordance with the human body and the inside components, including the brake pedal, throttle, steering wheel and gear are completely identical to a real vehicle (a Ford Focus). The driving simulator can also provide mimetic environmental noise and the equipped vibration simulation system and one degree of freedom motion platform can imitate the feeling of motion in order to largely replicate the realistic situations. This driving simulator has a 300-degree front view of display system with simulated back mirrors. Meanwhile, it also comes with a set of software to meet the requirements of driving scenario design, virtual traffic environment simulation and virtual road design, as shown in Figure 2.

### 2.2. Participants

This experiment recruited 50 healthy participants who were divided into two groups: 24 professional taxi drivers (15 males *vs.* 9 females) and 26 non-professional drivers (13 males *vs.* 13 females). The age of these drivers ranged from 20 to 52 (mean = 33.79, standard deviation = 9.95). Each participant had a valid driver's license, and the time since they were issued the first driver's license ranged from 1 to 25 years (mean = 9.04, standard deviation = 6.734). The specific descriptive statistics of participants is shown in Table 1.

### 2.3. Scenario Design

The Red-light-running (RLR) collision scenario in this experiment was defined as the pre-crash situation of potential collisions between the host vehicle (driving simulator) entering the intersection during the green phase and the other simulated RLR vehicle crossing the intersection from the right side of the driving simulator.

Five scenarios were designed in this paper with different combinations of warning status (yes or no), warning lead time to collision (3 s *vs.* 5 s) and warning content (with direction information *vs.* without), as shown in Table 2. Considering the time that drivers need to identify potential risk and take the corresponding collision avoidance maneuvers, the warning lead time design should ensure that the drivers have adequate brake reaction time and action time of collision avoidance for the RLR vehicle. In this study, 3 s is defined as late warning lead time and 5 s is defined as early warning lead time. The difference in the warning content is whether there is directional information provided in the audio information. The directional audio warning information was delivered as “alert to the red-light running vehicle from right side” while the non-directional warning information is “alert to the red-light running vehicle”.

Each subject needs to complete three rounds of tests for the five scenarios along an arterial road in the same simulated urban-environment road network, which is composed of a series of signalized intersections. The arterial road segment has one lane only per direction and its speed limit is 80 km/h. The intervals between adjacent intersections are 400 m. For each round of test drive, the signal at every intersection was always green along the driving route and the driver would encounter the unexpected RLR vehicle at one or two test intersections that were randomly selected in the network. The five scenarios were randomly assigned to the test intersections in order to counterbalance the temporal/spatial order effect during the experiment operation.

**Figure 3.**
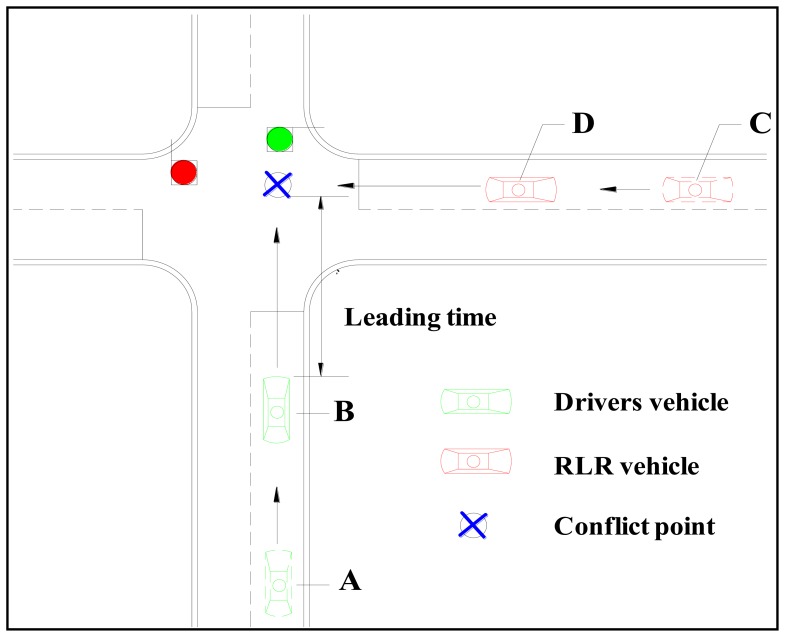
RLR pre-crash scenario design at the test intersections.

The RLR pre-crash scenario design at the test intersections is shown in Figure 3. In this study, the TTC sensors were used to monitor the approaching time of driving simulator to the conflict point at the intersection. When the approaching time was 7 s at the illustrated point A, the TTC sensor would trigger the RLR vehicle on point C started to cross the intersection with a velocity of 60 km/h. Because the approach time of the RLR vehicle to the conflict point was also designed as 7 s, the collision of these two vehicles will occur exactly at the conflict point as long as the velocity of the host vehicle did not change from the point A. Then, once the approaching time of driving simulator to the conflict point satisfies a predefined warning leading time value (3 or 5 s), the audio information will be released to alert the driver to the conflicting RLR vehicles.

### 2.4. Procedure

Upon the subjects arrival, the basic personal information such as age, gender, vocation and driving experience were collected first. Before the scenario tests, they were required to take a training process to become familiar with the basic operations of the simulator, including straight driving, acceleration, deceleration, left/right turn for at least 5 min until they adapted to the simulator operation. The training process also required the subjects to comply with traffic laws and behave just as they would normally drive in s real road environment. In the three formal experimental tests, each participant took a rest of 10 min between the two tests. In addition, the subjects may quit the experiment at any time if they feel uncomfortable or experience any motion sickness and their results from these suspended tests would be removed from the dataset.

### 2.5. Dependent Variables

Driving behaviors generally refer to drivers' responsiveness and control skills [23], including the abilities to regulate vehicle speed, control lane deviations and the response to potential hazards. In the HASTE research project [24], driving behaviors were characterized by the control ability of driving longitudinally and laterally. Specifically, the longitudinal control ability refers to the speed control and lateral control ability refers to the lateral lane deviation control. In order to analyze the impact of warning information on the effectiveness of collision avoidance, three parameters were defined in this study to reflect driving behaviors. The first parameter is brake response time (BRT) which is defined by the time between the appearance of RLR vehicle in the scenario (7 s approaching time to the conflict point) and the time that the host driver treads the brake pedal. It should be noted that BRT is a relative time to reflect how fast drivers will take actions to avoid collision based on the same time baseline, but not an absolute time value to measure drivers' reaction times after seeing the conflicting vehicle. The second parameter reflects the action of decelerating and defined by the maximum deceleration during the collision avoidance period from the time that the driver starts to tread the brake pedal to the time that avoidance was made or the driver passed the conflict point. The third parameter is the mean lane deviation during the collision avoidance process.

### 2.6. Structural Equation Modeling for Crash Avoidance Behavior Analysis

**Figure 4.**
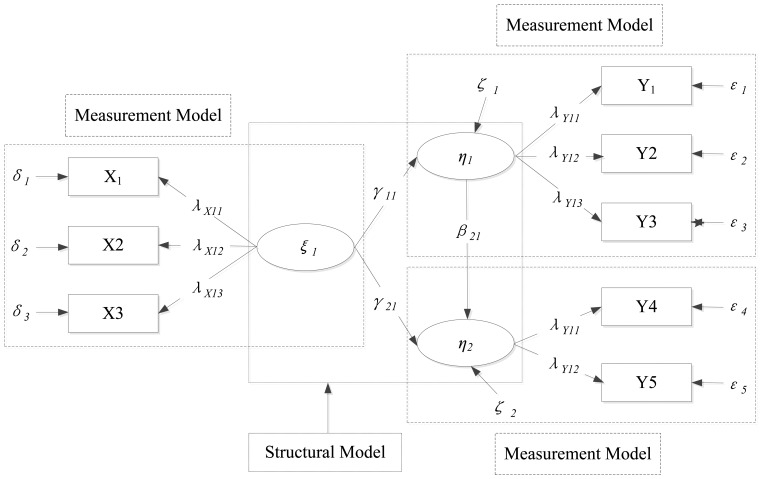
The basic example of SEM.

The previous analysis illustrates the relationships between warning conditions and the collision results. It also examines the impact of warning conditions on several measures of driving behaviors. Even so, it is still complicated and difficult to understand the underlying mechanism of collision avoidance because the traditional regression models are only capable to reveal simpler structure of relationship between the response and each explanatory variable, while the associations inspected by these models lack inference of causal explanations. Therefore, structural equation modeling (SEM) approaches which have been widely used in social science, economics, marketing and other research areas are gained more attentions in the transportation areas for understanding complicated causal relationships among important variables. A complete SEM consist two sub-models: measurement model and structural model. The measurement model describes the measured or operationalized relationships between latent variable and the manifest indicators which measure those latent variables, while the structural model aims at the causal relationship and weighting the influence between exogenous and endogenous variables [25]. A basic example of SEM is shown in Figure 4 with the definition of related symbols listed in Table 3 [26].

SEM indeed is a linear-in-parameter multivariate statistical modeling technique which combines factor analysis and simultaneous equation models. The relationship among observed variables, latent variables (measured or described by some manifest indicators) and disturbance variables/error variables can be examined, thus making it possible to capture the indirect, multiple, and reverse relationships among numerous variables [27]. Actually, as a variable can be an explanatory variable in one equation but a dependent variable in another equation, ‘endogenous’ variable and ‘exogenous’ variable are applied to differentiate them. In SEM graph, paths that are symbolized by arrows point toward exogenous variables and depart from the exogenous variables [28]. Endogenous variables can be influenced by exogenous variables directly or indirectly [29,30].

In this study, the SEM approach was adopted to further investigate the causal relationships between warning condition, human factors, driving behavior and the effectiveness of collision avoidance. The software package Analysis of Moment Structures (AMOS) 20 as part of SPSS was adopted to perform SEM analyses due to its advantage in exchanging data files completely from SPSS and the convenient graphical interface [31].

## 3. Results and Discussion

### 3.1. Collision Rate and Collision Severity

According to the results of experiments, the collision rate is calculated for different groups of participants in terms of their age, gender, vocation and other factors, as shown in Table 4. Among the 250 completed tests, 66 collisions happened and the remaining 184 trails successfully avoided the collisions. Therefore the overall collision rate in this experiment is 26.4%. Logistic regression analysis is then applied to investigate the impacts of independent variables on outcome of collision avoidance, and the hypothesis testing of the coefficients was based on a 0.05 significance level.

Age, gender, vocation, driving experience and warning information were used as independent variables in the logistic regression analysis and the results indicates that only warning information (P < 0.05) has a significant impact on the occurrence of collision. Thus, the final model contains only the variables of warning information and the estimation results are presented in Table 5. Generally, with the help of warning system, collisions are less likely to happen than the situation without any warning information. The result is in line with the study conducted by Suetomi *et al.* [32]. Referring to different warning lead times, a setting of 5 s has lower collision occurrence likelihood than the situation of 3 s. Further, the effects from combined characteristics of warning content and warning leading time are also different in resulting to the effectiveness of collision avoidance. Under 5 s warning lead time, the chance of collision is lower for the warning information with direction whereas if the warning lead time is 3 s, collisions are less likely to occur for the warning information without direction. For the longer warning lead time (5 s), drivers in host vehicles may have sufficient time to process the information. The warning information with information about the direction of the RLR vehicle can narrow the field of vision of a drivers' attention. In such a situation, the driver can quickly locate the RLR vehicle and take avoidance measures. However when the warning lead time is shorter (3 s), the warning information with direction may increase the driver's mental workload and delay the mental processing of the warning information.

### 3.2. Driving Behavior

#### 3.2.1. Brake Reaction Time

**Figure 5.**
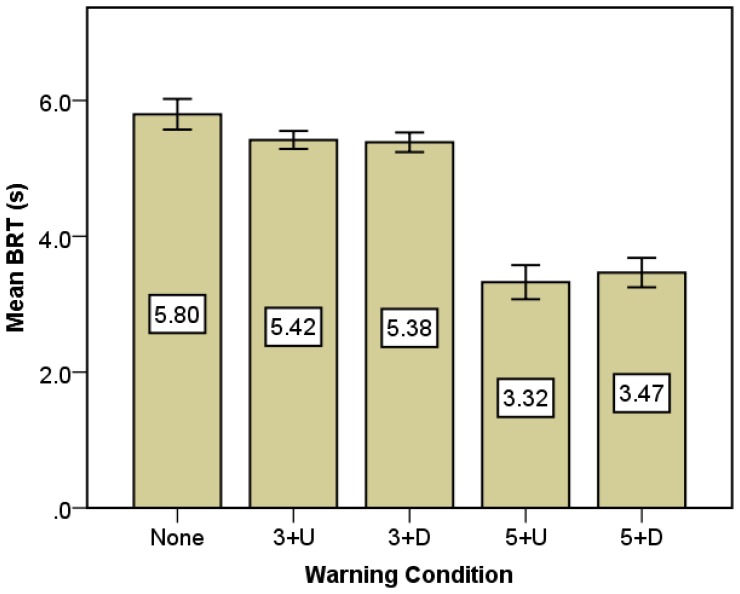
Mean BRT under different warning conditions.

Brake reaction time (BRT) was analyzed for the drivers taking brake action during the crash avoidance process in this study. It is observed that when the subjects encountered the conflicting RLR vehicle, three kinds of maneuvers were taken to avoid the potential crash occurrence, namely decelerating, accelerating and no reaction to the RLR vehicle. Among the 250 crash avoidance observations, there were in total 216 decelerating maneuvers, which are dominant behaviors in crash avoidance. The descriptive statistics of BRT among different types of drivers are reported in Table 6. In order to determine the impacts of personal characteristics, including age, gender, vocation, driving experience and audio warning information on driving behavior, the analysis of variance (ANOVA) model was conducted for BRT. Results provided in Table 6 show that only the warning condition has a significant impact (P < 0.001) on reaction time. Figure 5 also indicates that the participants without warning information took a longer time to respond to RLR vehicles than those with the warning information. It suggests that the audio warning information can effectively reduce the BRT to which in turn can assist drivers in avoiding collisions with RLR vehicles, which is consistent with the previous study [33]. In addition, BRT is much shorter for the situations with early warning lead time (5 s) comparing to the late warning lead time (3 s). Similar patterns have been discovered by Lee *et al.* [34]. Thus, early warning is advanced in terms of the shorter BRT [35]. However, respecting to the warning content, there is no significant difference between the warning information with and without direction.

#### 3.2.2. Maximum Deceleration

The basic descriptive analysis and ANOVA results of maximum deceleration are listed in Table 7. It is found that all the effects of age (P = 0.416), gender (P = 0.780), vocation (P = 0.952) and driving experience (P = 0.849) are insignificant, indicating the observed effects of these personal characteristics on deceleration cannot be discovered through this experiment. As the study aims at evaluating the effect of warning information on driving behavior, the comparison of maximum deceleration under different warning conditions is of the primary interest.

**Figure 6.**
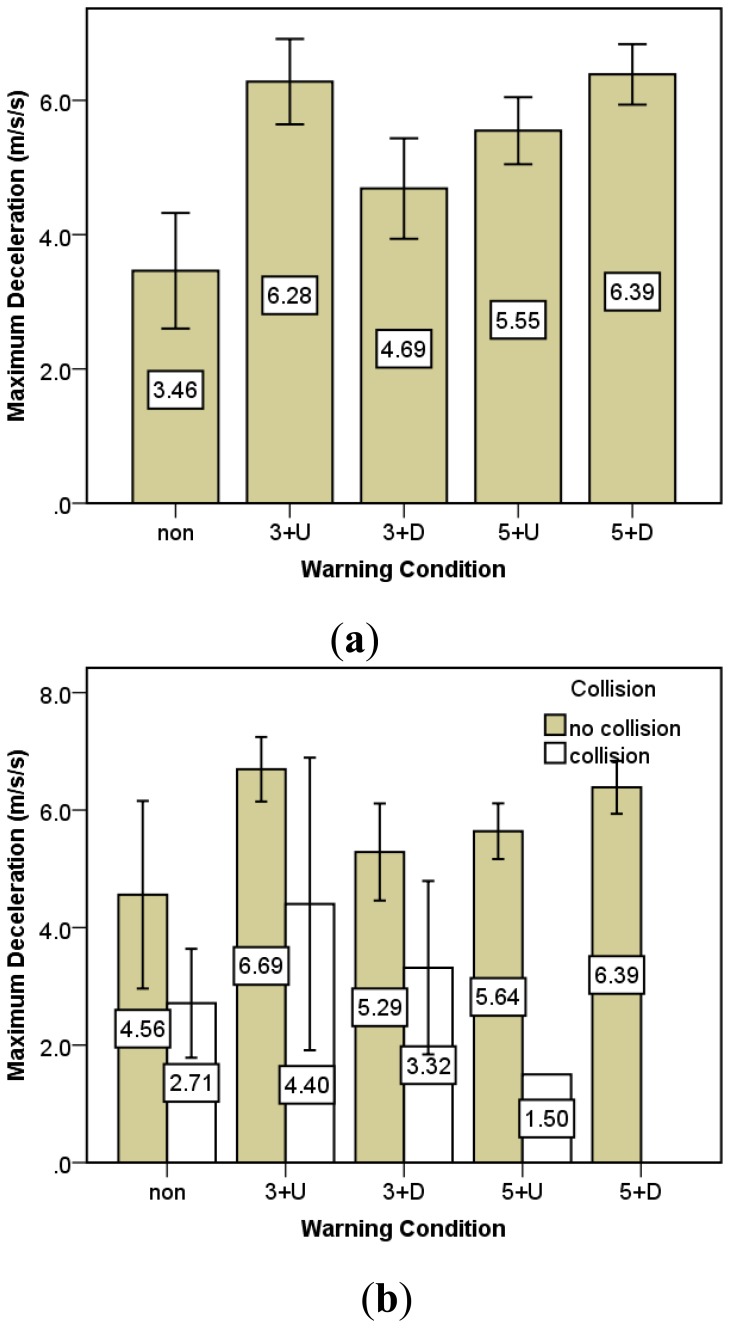
Maximum deceleration comparisons; (**a**) Under different warning conditions. (**b**) According to collisions and without collisions.

According to the results, warning conditions in fact have a significant impact on maximum deceleration (P < 0.001). Detailed maximum deceleration comparisons under five warning conditions are shown in Figure 6a. It indicates that the maximum deceleration in the cases without warning information was significantly smaller than in those with warning information. It also shows a trend that as the warning leading time increases, the maximum deceleration increases. Figure 6b further illustrates the results under five warning information conditions divided into groups of collision and no collision. It is found that compared to the collision group, the maximum decelerations applied by drivers in the no-collision group are consistently larger, indicating that essentially within a limited time and distance to the conflicting point, only the drivers who can slow down quickly can avoid collisions from the conflicting vehicle successfully. The maximum deceleration tends to be increased as the warning lead time is early and the warning content includes the directional hazard information. Interestingly, it is found that the deceleration rate for the 3 s undirectional warning condition is an exception from the other warning conditions, as it is much larger than the 3 s directional warning condition and even close to the 5 s directional warning condition. One possible reason is that because the 3 s warning leading time is quite late, drivers do not have sufficient time left to process the information when they receive the warning. Thus, a simple undirectional warning can advise drivers to brake harder more immediately than the directional warning, which may delay the mental processing of the warning information and cause drivers not to decelerate as much as possible. Referring to the result of collision occurrence rate, the 3 s lead time warning condition without direction information contributes to a lower collision rate than the 3 s lead time warning condition with direction information. Therefore, the pattern in the maximum deceleration is consistent with the result of collision occurrence rate.

#### 3.2.3. Lane Deviation

Lane control is another feature to reflect driving behavior and this study used the lane deviation from lane centerline as a measurement. Table 8 provides the descriptive statistics of departure distance and ANOVA results across other variables. It is observed that only the effect of gender (P = 0.030) and warning conditions (P = 0.055) are significant.

**Figure 7.**
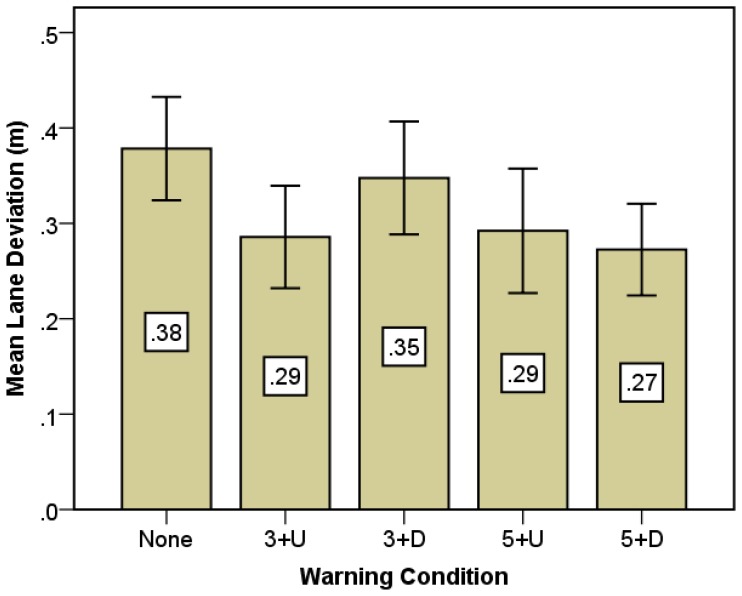
Mean lane deviation comparison under different warning conditions.

Figure 7 shows the mean lane deviations for different warning conditions. The lane deviation distance in the case of without warning is the largest among the five warning conditions. If drivers received the warning information in advance, they may have sufficient time to finish the deceleration, and thus do not need to rely on the steering wheel to avoid a collision. The horizontal deviation distance by steering was found to be smaller with early warning lead time compared to late warning lead time in general. The result indicates that the early warning can help drivers more confidently take a braking action to avoid collisions than the late warning. Additionally, for 3 s warning lead time, warning without direction information makes lane deviations smaller than the directional one (0.29 *vs.* 0.35 m). This was consistent with the maximum deceleration analysis as maximum deceleration with directional information is smaller than without in the case of the 3 s warning condition.

**Figure 8.**
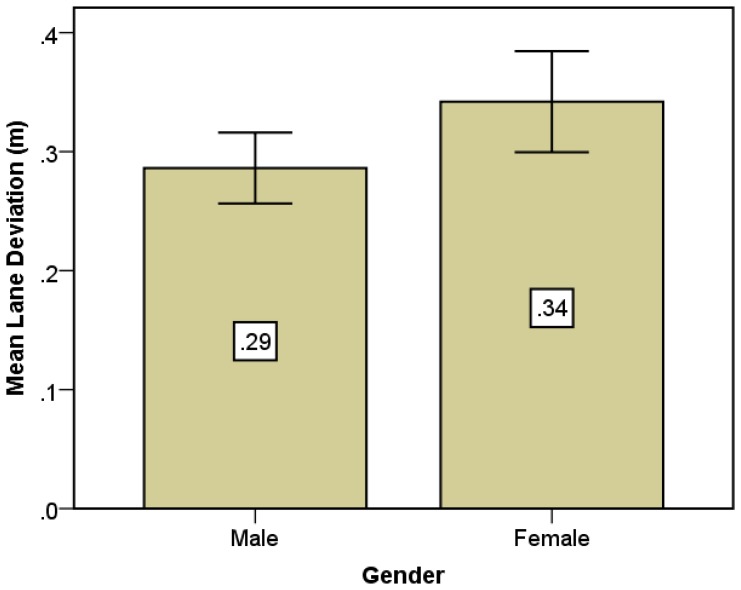
Mean lane deviation comparison between males and females.

Additionally, Figure 8 shows the difference was affected by gender. It was found that lane deviation of the female group was larger than the male one (0.34 *vs.* 0.29). The result suggests that the female drivers may panic more than male drivers in case of an emergency.

### 3.3. Analysis of Collision Avoidance Based on Structural Equation Modeling

**Figure 9.**
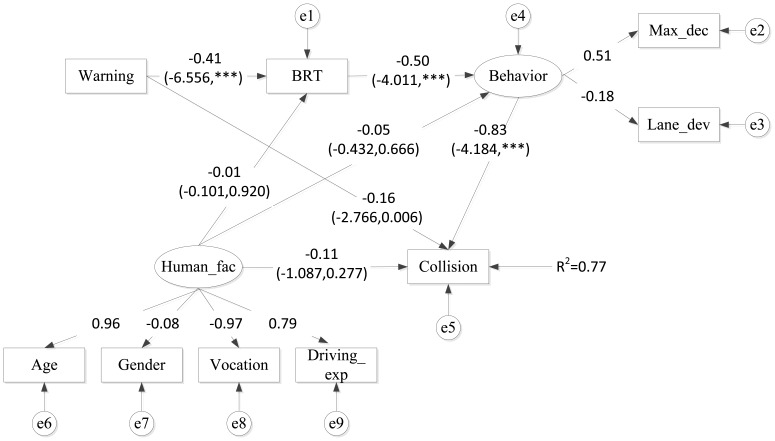
The SEM model.

According to the structure of SEM, the basic structural correlation among all the variables is shown in Figure 9, in which rectangle labels are used to represent observed variables while ellipse labels are used to represent latent variables. Measurement model is investigated with two important latent variables: person characteristics and driving behavior. In this model, the warning condition is an exogenous variable, driving behavior is an intermediate variable, and the final collision rate is the endogenous variable. Table 9 shows descriptions and input codes for the observed variables adopted in the following analysis.

In order to analyze the causal relationships between BRT and the other two variables of the driving behavior as well as the entire interaction among warning conditions, BRT and results of collision avoidance, a SEM was built for this purpose. In the SEM model, brake reaction time is assumed as a variable directly influenced by the warning conditions and that further affects the behavior. According to the output results of SEM model shown in Figure 8, it was found that the effect of warning on the final collision rates was divided into direct effect and indirect effect. Behavior is the most significant factor that negatively affects the collision (factor loading = −0.83, P < 0.001). Warning has a negative impact on BRT (factor loading = −0.41, P < 0.001), which indicates BRT can be decreased with warning information provided in the vehicle. Therefore, BRT was found to have a negative direct impact on behavior, suggesting shorter BRT leads to larger maximum deceleration and smaller lane deviation indirectly. It therefore implies that shorter BRT would lead to drivers' action of decelerating and lateral lane control, and larger maximum deceleration and smaller lateral lane deviation are able to effectively decrease the chance of collisions with RLR vehicles. In addition, warning also has a direct effect on collision occurrence (factor loading = −0.16, P = 0.006). Compared to the indirect effect of warning on collision occurrence (from warning to BRT to behavior then to collision), the most impact of warning condition on final collision occurrence or not was mostly contributed by the indirect effect. Finally, with the warning information, chance of collisions can be greatly reduced, which is consistent with the previous studies [32,34].

However, personal characteristics is an exogenous variable and has no direct effect on BRT and collisions according to the model (factor loading = −0.01, P = 0.920; factor loading = −0.11, P = 0.277 respectively). Although different attributes such as age and gender may lead to different behaviors, the observed differences in terms of BRT and driving behaviors were relatively small in magnitude. Such a finding is in fact consistent with the previous study [36]. On the whole, the SEM model explained 77% of total variance in the results of collision avoidance.

In order to examine data fitting performance, a widely series of Goodness of Fit criteria were adopted in the structural equation models. If the model fits the data well, the Chi-square value should be smaller and p > 0.05 [37,38]. However, the Chi-square value is inclined to increase if the sample size grows [28,39]. As a result, several alternative criteria are commonly applied to examine the fitness of model. Referring to prior studies, the conditions that CMIN/DF < 2, GFI, AGFI, CFI and NFI are all greater than 0.9, or RMSEA smaller than 0.1, also indicate that the model has an acceptable fitness with the data [26,40]. Accordingly, the model adopted in this study can reach an acceptable fitness level. Table 10 presents these criteria for measuring the goodness of fit in this model.

## 4. Conclusions

Technologies for effectively reducing traffic collision are important. Considering the rapid progress in the area of information technologies, audio warning information is suggested to address this issue. However, the causal relationships between warning information and driving behavior, as well as the correlation between driving behavior and occurrence of collisions are still ambiguous and require further understanding on the influencing mechanism(s). This study focused on audio warning information which is delivered to remind drivers who are approaching intersections during the green phase to be alert to conflicting illegal RLR vehicles. A high-fidelity driving simulator was used to test the effectiveness of the warning system under different warning lead times and warning contents.

A comparison between the conditions with warning information and without warning information shows that audio warning information has an explicit positive effect on reducing collision rates. The collision rates under the two conditions are 66% and 16.5%, respectively for with-warning and without-warning. As a result, RLR collisions can be dramatically decreased by 75% if warning information is presented. The experiment results also indicated there was a tendency that the warning information leads to shorter BRT, larger deceleration and less lane deviation compared to the no-warning condition. In comparison between different warning lead times, early warning has a better effect on collision avoidance as lower collision occurrence likelihood, shorter BRT, larger deceleration and less lane deviation were found. As for warning content, it was found that there was no significant difference in drivers' crash avoidance behavior and result between directional and undirectional information for the early warning. However, an interesting finding is that for the late warning conditions, the effect of undirectional information on collision avoidance was better than the directional information. It implies that in a very emergent situation, the advantage of simple warning content over complex one is helping drivers reduce mental workload and take timely crash avoidance actions.

Furthermore, the casual relationships between warning information and the results of collision avoidance were investigated by a SEM model. The findings reveal that warning information has both direct and indirect impacts on collision occurrence. The indirect impact produced by affecting BRT behavior then to the collision is much larger than the direct effect. The SEM model demonstrated that shorter BRT leads to larger deceleration and smaller lane deviation that caused a lower collision rate. In the examination of personal characteristics, although drivers' personal attributes are different, results also show that these personal characteristics do not affect BRT or driving behaviors significantly. Therefore, the occurrence of collisions may not be influenced by these personal characteristics (e.g., age and gender).

In conclusion, before identifying and developing the mature vehicle-based and infrastructure-based technologies to be applied to intersections, driving simulators can serve as a useful tool to test these technology concepts, investigate human factors involved in the application of these technologies, and evaluated the effectiveness of the systems on driver behavior and intersection safety. This study conducted an interactive simulation to test the effectiveness of function and mechanism of in-vehicle speech collision avoidance warning system. The scenario design and simulation results are helpful to direct researchers in better developing and realizing the relevant sensors-related technologies in practice. The experimental results show that audio warning information has a positive effect on reducing the RLR collision rate at intersections.

Based on the findings in this study, some further works have been identified for the near future. Firstly, drivers' psychological and physiological measures were not collected and analyzed in this study. The collection of data from eye trackers and EEG instruments is suggested to examine drivers' cognitive behaviors and physiological variations under different warning conditions. Secondly, this experiment only designed two levels of warning lead times (3 s *vs.* 5 s), which clearly indicated that the early warning is more effective on crash avoidance than the late warning. However, the more early the warning time is, the greater the possibility that the warning system will make a false predication of crash occurrence. It is suggested that more warning leading times should be tested to explore the optimal warning time in order to balance safety effectiveness of warning and power of crash prediction. Finally, if test vehicles and sites are available, under the assumption that the subjects' safety can be ensured, a field study is suggested to test the functions of the warning system and validate the experimental results of this driving simulator experiment.

## Figures and Tables

**Table 1.** Descriptive statistics of participants.

**Driver Characteristics**	**Classification**	**Driver Numbers**	**Proportion**
Age	Young (20–30 years old)	24	48.0%
	Middle-aged (30–52 years old)	26	52.0%
Gender	Male	28	56.0%
	Female	22	44.0%
Vocation	Professional drivers	24	48.0%
	Non-professional drivers	26	52.0%
Driving experience	Primary (1–3 years)	13	26.0%
	Middle (4–9 years)	17	34.0%
	Senior (≥10 years)	20	40.0%

**Table 2.** Experimental scenarios based on different warning conditions.

**Intersection**	**Warning Status**	**Warning Strength**	**Warning Lead Time**
1	Warning	With direction	3 s
2			5 s
3		Without direction	3 s
4			5 s
5	Without warning	none	none

**Table 3.** Definition of symbols in Figure 4.

	**Symbol**	**Definition**
Measurement model	X	q × 1 column vector of observed variable or manifest indicator of *ξ*
	Y	p × 1 column vector of observed variable or manifest indicator of *n*
	*ξ*	n × 1 column vector of latent exogenous variables
	*n*	m × 1 column vector of latent endogenous variables
	*δ*	q × 1 column vector of measurement error terms for observed variables X
	*ε*	p × 1 column vector of measurement error terms for observed variables Y
Structural model	*γ*	The matrix (m × n) of regression effects for exogenous latent variables to endogenous latent variables
	*β*	The coefficient matrix (m × m) of direct effects between endogenous latent variables
	*ζ*	m × 1 column vector of the error terms

**Table 4.** Descriptive statistics of collisions in experiments.

**Driver** **Characteristics**	**Classification**	**Collision**				**Total** **Count**

		**No**		**Yes**		

		**Count**	**Row N%**	**Count**	**Row N%**	
Age	Young (20–30 years old)	87	72.5%	33	27.5%	120
	Middle-aged (30–52 years old)	97	74.6%	33	25.4%	130
Gender	Male	109	77.9%	31	22.1%	140
	Female	75	68.2%	35	31.8%	110
Vocation	Professional	91	75.8%	29	24.2%	120
	Non-professional	93	71.5%	37	28.5%	130
Driving experience	Primary (1–3 years)	45	69.2%	20	30.8%	65
	Middle (4–9 years)	63	74.1%	22	25.9%	85
	Senior (≥10 years)	76	76.0%	24	24.0%	100
Warning conditions	Without	17	34.0%	33	66.0%	50
	3 s without direction	38	76.0%	12	24.0%	50
	3 s with direction	33	66.0%	17	34.0%	50
	5 s without direction	47	94.0%	3	6.0%	50
	5 s with direction	49	98.0%	1	2.0%	50
Total		184	73.60%	66	26.40%	250

**Table 5.** Results of the logistic regression analysis.

**Warning Status**	**B**	**S.E.**	**Wald**	**Df**	**Sig.**	**Exp(B)**
Constant	–3.892	1.010	14.843	1	0.000	0.020
Without warning	4.555	1.053	18.701	1	0.000	95.118
3 s without direction	2.739	1.063	6.639	1	0.010	15.474
3 s with direction	3.229	1.053	9.394	1	0.002	25.242
5 s without direction	1.140	1.173	0.946	1	0.331	3.128
5 s with direction	——	——	44.389	4	0.000	——

**Table 6.** The descriptive statistics of BRT and ANOVA results.

**Independent** **Variable**	**Classification**	**Reaction Time**					**ANOVA** **P-value**

		**Mean**	**Count**	**Min**	**Max**	**S.D.**	
Age	Young (20–30 years old)	4.60	107	6.50	2.10	1.28	0.845
	Middle-aged (30–52 years old)	4.57	109	6.50	2.10	1.20	
Gender	Male	4.63	120	6.50	2.30	1.22	0.578
	Female	4.54	96	6.50	2.10	1.27	
Vocation	Professional	4.56	101	6.50	2.10	1.21	0.718
	Non-professional	4.62	115	6.50	2.10	1.26	
Driving experience	Primary (1–3 years)	4.67	58	6.50	2.10	1.38	0.838
	Middle (4–9 years)	4.58	75	6.50	2.10	1.18	
	Senior (≥10 years)	4.54	83	6.50	2.40	1.20	
Warning conditions	Without	5.80	32	6.50	4.20	0.62	<0.001
	3 s without direction	5.42	44	6.40	4.20	0.43	
	3 s with direction	5.38	46	6.50	4.40	0.49	
	5 s without direction	3.32	45	5.20	2.10	0.84	
	5 s with direction	3.47	49	5.70	2.10	0.76	

**Table 7.** The descriptive statistics for deceleration and ANOVA results.

**Independent** **Variable**	**Classification**	**Maximum Deceleration**					**ANOVA** **P-value**

		**Mean**	**Count**	**Max**	**Min**	**S.D**	
Age	Young (20–30 years old)	5.52	107	8.45	0.08	2.26	0.416
	Middle-aged (30–52 years old)	5.27	109	10.38	0.11	2.30	
Gender	Male	5.43	120	10.38	0.08	2.32	0.780
	Female	5.35	96	8.45	0.26	2.24	
Vocation	Professional	5.40	101	10.38	0.11	2.22	0.952
	Non-professional	5.39	115	8.45	0.08	2.34	
Driving experience	Primary (1–3 years)	5.33	58	8.31	0.35	2.18	0.849
	Middle (4–9 years)	5.52	75	10.38	0.08	2.43	
	Senior (≥10 years)	5.33	83	8.54	0.11	2.22	
Warning condition	Without	3.46	32	7.74	0.08	2.39	0.000
	3 s without direction	6.28	44	8.52	0.33	2.09	
	3 s with direction	4.69	46	10.38	0.11	2.52	
	5 s without direction	5.55	45	8.46	1.50	1.66	
	5 s with direction	6.39	49	8.54	2.55	1.57	

**Table 8.** Descriptive statistics of lane deviation characteristics and ANOVA results.

**Independent** **Variable**	**Classification**	**Lane Deviation**					**ANOVA** **P-value**

		**Mean**	**Count**	**Max**	**Min**	**S.D.**	
Age	Young (20–30 years old)	0.31	107	0.74	0.01	0.18	0.768
	Middle-age (30–52 years old)	0.31	109	0.84	0.00	0.20	
Gender	Male	0.29	120	0.72	0.01	0.16	0.030
	Female	0.34	96	0.84	0.00	0.21	
Vocation	Professional	0.32	101	0.84	0.00	0.20	0.613
	Non-professional	0.30	115	0.74	0.01	0.18	
Driving experience	Primary (1–3 years)	0.30	58	0.74	0.00	0.17	0.189
	Middle (4–9 years)	0.29	75	0.66	0.01	0.18	
	Senior (≥10 years)	0.34	83	0.84	0.01	0.20	
Warning condition	Without	0.38	32	0.75	0.10	0.15	0.055
	3 s without direction	0.29	44	0.66	0.02	0.18	
	3 s with direction	0.35	46	0.84	0.00	0.20	
	5 s without direction	0.29	45	0.82	0.01	0.22	
	5 s with direction	0.27	49	0.66	0.01	0.17	

**Table 9.** Definition of variables in SEM models.

**Observed Variables**		**Coding of Input Value**

**Name**	**Description**	
Age	Age group	0→Young 1→Middle-aged
Vocation	Participants' vocation	0→Professional 1→Unprofessional
Driving_exp	Driving experience	Continuous variable
Gender	Gender	0→Male 1→Female
Warning	Warning or not	0→Without warning 1→Warning
Max_dec	The maximum deceleration during decelerating process	Continuous variable
Lane_dev	Lateral position deviation distance to avoid the RLR vehicle	Continuous variable
BRT	Time between warning messages released and driver take a brake	Continuous variable
Collision	Whether there is a collision between two cars	0→No 1→Yes

**Table 10.** Fit statistics for SEM models.

**Fit Index**	**Acceptable Fit**	**Model**
CMIN/DF	<2	1.559
P-value	>0.05	0.057
GFI (Goodness of Fit Index)	>0.9	0.973
RMSEA (Root Mean Square Error of Approximation)	<0.1	0.051
AGFI (Adjusted Goodness of Fit Index)	>0.9	0.937
CFI (Comparative Fit Index)	>0.9	0.987
NFI (Normed fix index)	>0.9	0.965
